# Inferring archaic introgression from hominin genetic data

**DOI:** 10.1002/evan.21895

**Published:** 2021-05-05

**Authors:** Shyamalika Gopalan, Elizabeth G. Atkinson, Laura T. Buck, Timothy D. Weaver, Brenna M. Henn

**Affiliations:** 1 Department of Ecology and Evolution, Stony Brook University, Stony Brook, New York; 2 Department of Evolutionary Anthropology, Duke University, Durham, North Carolina; 3 Analytic and Translational Genetics Unit, Massachusetts General Hospital and Stanley Center for Psychiatric Research, Broad Institute, Boston, Massachusetts; 4 Research Centre in Evolutionary Anthropology and Palaeoecology, Liverpool John Moores University, Liverpool, UK; 5 Department of Anthropology, University of California, Davis, California; 6 UC Davis Genome Center, University of California, Davis, California

**Keywords:** ancient DNA, archaic hominins, genetics, introgression

## Abstract

Questions surrounding the timing, extent, and evolutionary consequences of archaic admixture into human populations have a long history in evolutionary anthropology. More recently, advances in human genetics, particularly in the field of ancient DNA, have shed new light on the question of whether or not *Homo sapiens* interbred with other hominin groups. By the late 1990s, published genetic work had largely concluded that archaic groups made no lasting genetic contribution to modern humans; less than a decade later, this conclusion was reversed following the successful DNA sequencing of an ancient Neanderthal. This reversal of consensus is noteworthy, but the reasoning behind it is not widely understood across all academic communities. There remains a communication gap between population geneticists and paleoanthropologists. In this review, we endeavor to bridge this gap by outlining how technological advancements, new statistical methods, and notable controversies ultimately led to the current consensus.

## 1 | INTRODUCTION

During the 1980s and 1990s, several models of modern human origins were vigorously debated by paleoanthropologists. At the extremes of the spectrum were the multiregional and recent African origin (RAO) models. The multiregional model proposed significant continuity between *anatomically modern humans* (AMH) and “archaic” progenitors in different regions of Eurasia and Africa. According to this view, *H. sapiens* originated over 1 million years ago and speciation between regional subpopulations was prevented by substantial gene flow.^1,2^ The RAO model describes an exclusively, and relatively recent, African origin for *H. sapiens*, with subsequent global dispersal and rapid replacement of other hominin taxa at around 50,000–60,000 years ago (ka).^3–5^ Intermediate between these extremes were models such as Bräuer’s “hybridization and replacement” model, which posits an African origin, but allows for gene flow between African-derived *H. sapiens* and other hominin taxa during dispersals.^6,7^ Likewise, Smith’s and Trinkaus’ assimilation models^8,9^ are variations on the multiregional model in that they emphasize substantial and widespread gene flow between *H. sapiens* and other groups while acknowledging the central role of Africa as the primary birthplace of the species (Box 1).

Results from human genetic data began to weigh in on these debates in 1987, when a survey of the D-loop section of the *mitochondrial genome* of 147 people from extant modern human populations supported a recent African origin based on the limited genetic differences among individuals from different continents, with the basal lineages being carried by Africans.^10^ More mitochondrial DNA (mtDNA),^11^ and later Y chromosome data,^12^ from extant human populations corroborated a RAO, as *coalescence* times among sequences were dated to 50–200 ka, indicating a very recent common ancestry for all extant humans.^11,12^ Subsequently, genome-wide *autosomal DNA* further corroborated the RAO hypothesis.^13^ By the mid-1990s, geneticists generally agreed that modern human DNA showed no evidence for introgression from Neanderthals or other archaic hominins into AMH,^14^ even though some models indicated that the observed data could be consistent with small amounts of gene flow (Table 1; Box 2).

In the last decade, the majority opinion of geneticists has reversed; today, it is broadly agreed that there have been multiple introgression events from archaic groups into AMH populations. This abrupt reversal is perhaps the most notable in the young field of human evolutionary genetics. Here, we review the genetic literature that led up to the current state of the field, focusing on the methodological aspects of archaic admixture inference, primarily from aDNA, in the context of population genetic theory. We present estimates of the average archaic content of modern human populations from the literature, as well as the estimated timing and location of the introgression events, while emphasizing how various methods and different assumptions about demographic history can affect the conclusions that are drawn. In doing so, we aim to provide general guidelines for non-specialist audiences to evaluate the human genomics literature pertaining to archaic introgression.

## 2 | ESTIMATES OF ARCHAIC ADMIXTURE BASED ON ANCIENT MITOCHONDRIAL DNA SEQUENCING

**FIGURE 1:**
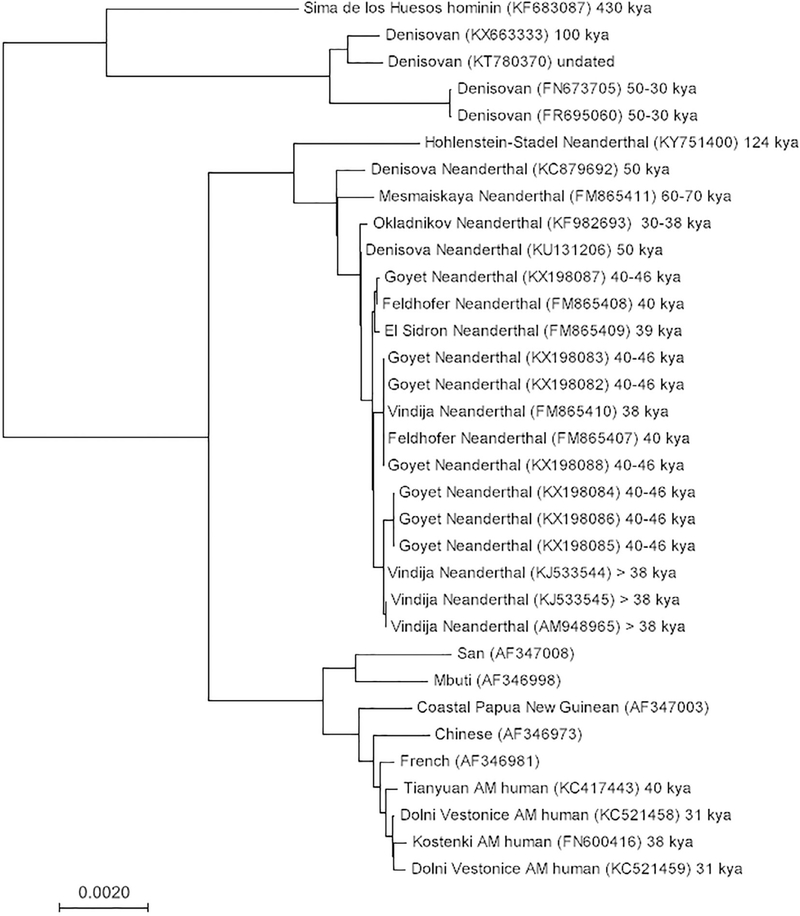
The mitochondrial phylogeny of a Sima de los Huesos hominin, four Denisovan, 19 Neanderthal, 5 extant human, and 4 ancient AMH mitochondrial sequences (15,788 aligned base pairs in total) constructed using the neighbor joining method.^120,121^ The branch lengths are proportional to the evolutionary distances computed using maximum composite likelihood. All analyses were conducted in MEGA7.^122^ Branch tips are labeled with a sample name, the accession number of the downloaded sequence in brackets, and the approximate date of the specimen.^32,35,75,123–136^ The tree shows that Neanderthal mitochondrial sequences are more highly diverged from extant humans than all AMH (ancient and extant) are from each other. Interestingly, the mitochondrial phylogeny places Neanderthals and AMHs as sister groups to the exclusion of Denisovans and the Sima de los Huesos hominin, as observed previously.^136^ This is in contrast to the phylogeny constructed from multiple loci of autosomal DNA, which instead places Neanderthals and Denisovans as sister groups.^35^ This discrepancy highlights the fact that inferences of population history based on single loci can be misleading, as they reflect the history of only one gene lineage

The first ancient Neanderthal DNA to be successfully isolated and analyzed was mtDNA from the Neander Valley type specimen, discovered in 1856.^15,16^ The relative abundance of mtDNA in cells, compared with nuclear DNA, made it a logical starting place for sequencing ancient Neanderthal DNA (Box 2). These first studies found that, across a total of 600 base pairs (bp) of sequence, Neanderthal mtDNA fell well outside the bounds of extant human mtDNA variation (Figure 1), exhibiting on average three times as many pairwise differences from extant humans as different human populations did between each other. Importantly, these researchers also did not find that Neanderthal mtDNA was more similar to that of Europeans than to that of Africans or Asians. This observation went against a key prediction under the multi-regional model that Neanderthals contributed substantially to the ancestral gene pool of modern Europeans.^2,17,18^ Sequence differences were also used as a molecular measure of divergence time, calibrated using a human–chimpanzee divergence of 4–5 million years ago.^19,20^ Both studies consistently found a mtDNA *sequence divergence* time of approximately half a million years between the Neander Valley specimen and modern humans, which is approximately three to four times older than the average divergence time between extant human mtDNA sequences (Figure 1).^15,16^ These results from a single Neanderthal showed that its mtDNA was evolving separately from AMHs for over half a million years and is no longer present in the modern human gene pool.

Additional work expanded these analyses by including mtDNA sequence data from an additional Neanderthal individual from Vindija Cave, Croatia.^21^ This individual’s mtDNA also exhibited large sequence differences from extant human mtDNA sequences, and phylogenetic analysis placed these Neanderthals together in a deeply diverged clade.^21^ The degree of sequence diversity of the Neanderthal population was estimated by comparing the two sequences to each other and to a third shorter mtDNA sequence from a more ancient Neanderthal individual from Mezmaiskaya Cave, Russia.^21^ By sequencing multiple archaic individuals, especially ones so geographically dispersed, researchers could confidently say that Neanderthal mtDNA sequences were highly distinct from those of modern humans and were not more closely related to any one extant population. Furthermore, mitochondrial aDNA sequences from nearly contemporaneous Upper Paleolithic AMH specimens were found to fall within the range of modern human mtDNA variation, distant from the Neanderthals.^22,23^ The presence of significant genetic differences between the mtDNA of AMH and Neanderthal groups that lived within just 15 ka of each other implied strong reproductive boundaries between the two groups, and contradicted the classic multiregional hypothesis.

The analysis of mtDNA led most geneticists to initially conclude that archaic introgression did not occur.^22,24,25^ The availability of additional mtDNA sequencing data has also not significantly changed the broad phylogenetic pattern (Figure 1). However, mtDNA is a single *locus*, and can therefore offer only limited information about potential archaic admixture (Box 2). Non-neutral forces such as natural selection for AMH mtDNA (or against archaic mtDNA) could have also led to the complete loss of Neanderthal mitochondrial variation in AMH.^26^ Another possibility is that the interbreeding event(s) were sex-biased; in the extreme case, where 100% of interbreeding events involved a Neanderthal male and an AMH female, Neanderthal mtDNA would have never entered the modern human gene pool. Additionally, *genetic drift* could have erased evidence of archaic introgression from the extant pool of mitochondrial variation. Several population genetics models showed that some degree of interbreeding is compatible with an absence of archaic mtDNAs in the modern gene pool (Table 1). These various models were, however, difficult to test further without information from additional independent loci, such as from the *nuclear genome*. Despite these data limitations, geneticists generally agreed that archaic-modern human matings were an unlikely (or at least infrequent) occurrence, a consensus that held until the first archaic hominin nuclear DNA sequencing results were published in 2006.

## 3 | ARCHAIC AUTOSOMAL GENOMES

It was not always obvious that the full nuclear genome of an archaic individual would ever be sequenced. aDNA, if it survives in any appreciable quantity, is highly damaged and fragmented, which makes piecing long sequences together a major technological and computational challenge. However, the development of “next-generation sequencing” (NGS) technology in the 2000s significantly mitigated this problem. One benefit of NGS is that individual loci do not need to be specifically targeted to be sequenced; it is capable of sequencing a random selection of all the fragments in a DNA sample. The resulting short reads can later be assembled computationally by *mapping* (or aligning) them to a reference genome. By contrast, the Sanger sequencing method used in earlier studies required researchers to specify a genomic target region. This can only be accomplished if the entire region plus its flanking sequences are intact in the ancient sample. While Sanger sequencing had the capability of producing longer reads (up to 1,000 bp), this benefit was not often realized in aDNA studies where input DNA is typically already in fragments of 10s to 100s of bp long. On the other hand, NGS was designed to produce large quantities of short reads in parallel, which had the additional effect of driving down the sequencing cost per base.

These technological advances re-opened the possibility of ancient genome sequencing, and in 2006 two competing research groups, who produced articles authored by Green et al. (from the Max Planck Institute for Evolutionary Anthropology) and Noonan et al. (from the Joint Genome Institute), published analyses of large amounts of Neanderthal nuclear sequence.^27,28^ In these studies, researchers independently extracted, sequenced and analyzed DNA from the same Neanderthal specimen from Vindija, Croatia. However, there were major inconsistences between the results of these two studies (Box 3), which called their validity into question.^29^ While each study utilized different sequencing technologies, analytical methods, and genomic regions, these factors were not sufficient to account for the magnitude of the discrepancies in the results. In order to understand these inconsistencies, Wall and Kim re-analyzed both datasets using a uniform set of analytical methods, and concluded that the dataset used in the Green et al. study had been significantly contaminated by modern human sequences (Box 3).^29^ This was later confirmed by some of the authors of the original Green et al. article, who estimated that their Neanderthal dataset contained between 11 and 41% contamination.^30^

This case study serves to highlight the difficulties of working with hominin aDNA.^31^ Relatively small amounts of modern human contamination, such as from the scientists and archeologists handling the ancient specimens, can overwhelm the scant, fragmented amounts of authentic aDNA, and end up accounting for a large proportion of the total DNA sourced from a specimen. Contamination from modern sources is often impossible to eliminate completely, even when the best laboratory practices are in place. Ancient specimens have often been handled without safeguards against DNA contamination in mind, as many were discovered and excavated decades ago. Fortunately, improved understanding of DNA damage patterns, their time dependency, as well as new bioinformatic methods have made sequence data from even significantly contaminated libraries useful for analysis.^32,33^ The increased awareness of the problem of modern contamination has certainly improved the quality of DNA studies of ancient hominins. Nevertheless, critical assessment of such studies will continue to be important, in particular by considering them in the context of the archeological record and existing genetic studies.

## 4 | ESTIMATING THE FRACTION OF ARCHAIC ANCESTRY IN MODERN HUMAN GENOMES

In 2010, following their initial attempt to sequence Neanderthal autosomal DNA, researchers at the Max Planck Institute for Evolutionary Anthropology produced a full, low *coverage*, Neanderthal genome.^34^ The researchers produced this genome by combining sequencing data from three Neanderthal individuals from Vindija.^34^ Importantly, they explicitly estimated contamination levels of their libraries. By looking at diagnostic positions in the mitochondrial genome where Neanderthals and modern humans carried fixed differences, Green et al. concluded that contamination by modern humans contributed less than 1% to their dataset.^34^

The most highly publicized result of this 2010 article was that individuals from certain extant human populations contain a substantial amount, between 1 and 4%, of Neanderthal-derived ancestry in their genomes (Table 2).^34^ The researchers arrived at this figure by developing a novel test, which came to be known as the “D” or “ABBA-BABA” test (Box 4). This test calculates the “D statistic,” and is based on the idea that if a human population experienced archaic introgression in the past, it will exhibit greater genetic similarity to the archaic population than one that has not. In order to detect this, Green et al. examined *single nucleotide polymorphisms (SNPs)* where the archaic genome shared the derived *allele* with one human genome, while another human genome carried the ancestral allele (defined as the allele carried by the chimpanzee).^34^ If there had been no archaic introgression into either human population’s ancestors, neither genome should exhibit an excess of allele sharing with the Neanderthal. This would also occur if equal amounts of introgression occurred in all human populations, but it was assumed that ancestral sub-Saharan African populations did not mate with Neanderthals due to the fact that their ecological and geographic ranges did not overlap. The initial results of this test indicated that the Neanderthal was more similar to modern individuals of European and East Asian ancestry than to individuals of African ancestry, consistent with Neanderthal introgression into the ancestors of all Eurasians.^34^

The D-statistic approach was designed to leverage the limited information that could be derived from a single, low coverage genome that was actually a “mosaic” of three distinct individuals. It provided a parameter that could be used in an explicit population genetic model to estimate the percentage of modern non-African genomes that is of Neanderthal origin. The model in Green et al. also included parameters for the sizes of the ancestral populations, their *population divergence* times, and when Neanderthals and non-African AMH last exchanged genes. Under this model, the researchers determined that the range of *f*, the proportion of a modern genome that is of Neanderthal origin, most compatible with the observed D-statistics is 1–4% for non-African individuals. It is important to note that this model is extremely simplified in order to be mathematically tractable. It assumes a single, discrete episode of gene flow from Neanderthals to humans, completely panmictic ancestral populations, and does not consider the possibility of population growth or genetic drift over time.

Green et al. used a second method to derive a direct estimate of *f* using the ratio between un-normalized D-statistics, or S-statistics (Box 4). This method estimates the percentage of Neanderthal ancestry in modern non-African genomes to be 1.3–2.7%, which is in general agreement with results obtained from their population genetic model. By using these and other strategies, Green et al. concluded that AMH did interbreed with Neanderthals in the relatively recent past.^34^ However, they could not entirely rule out the possibility that other demographic scenarios could have produced the observed genetic patterns in the complete absence of introgression. Specifically, with respect to its effect on D-statistics and S-statistics, ancestral population structure is expected to mimic the signature of archaic introgression (see section on “Alternative explanations”).

Soon after the publication of the draft Neanderthal genome, a second archaic genome of an individual from the Altai mountains was published.^35^ This individual was sequenced from a single finger bone, and was found to be genetically divergent from both modern humans and Neanderthals. Nuclear sequence data placed this group as sister to Neanderthals.^35^ The specimen was designated as a member of an unknown archaic population, which was named “Denisovan” after Denisova cave in Siberia where it was discovered.^35^ As in the Neanderthal study, the researchers estimated *f*, the proportion of Denisovan ancestry in modern humans, using both parametric and non-parametric approaches. Interestingly, they found a large contribution (4–6%) of this archaic group to modern Melanesians, but no contribution to Eurasians (Table 3).^35^ Subsequent research has estimated the Denisovan contribution to Melanesians to be only about half that, after also accounting for Neanderthal admixture.^36,37^ Additional studies have used a variety of methods to estimate *f* in both Neanderthals and Denisovans; a summary of these estimates is found in Tables 2–3. In general, initial estimates of the archaic fraction of modern human genomes have tended to be high, with later publications revising these figures downward.

## 5 | HAPLOTYPE-BASED METHODS TO IDENTIFY GENOMIC REGIONS OF INTROGRESSION

Following the publication of the low coverage Neanderthal genome sequence, researchers began to highlight specific loci where some modern humans carried *haplotypes* that were hypothesized to have an archaic source, uncovering evidence for introgression on a finer genomic scale.^38,39^ Even before nuclear data from archaic hominins were available, haplotype analyses of modern humans were used to identify genomic candidates of archaic introgression.^40–43^ These methods took the general approach of looking for haplotypes that were both highly diverged from other modern humans and also relatively long (Box 5). Once high coverage archaic genomes became available, some of these cases were re-evaluated by comparing the hypothesized archaic haplotypes to their putative ancestral source.

In one study, Yotova et al. studied a specific haplotype on the X chromosome that is nearly absent in sub-Saharan Africans, common in non-Africans, and the most basal human haplotype.^39^ Such a pattern is unexpected under a RAO model with no archaic introgression, although not impossible (see section on “Alternative explanations”). Yotova et al. also found that this haplotype was similar to the published Neanderthal sequence, leading them to conclude that it entered the modern human gene pool through introgression.^39^ Mendez et al. used a comparable approach to suggest that a specific haplotype of *STAT2*, a gene involved in immune function, introgressed from Neanderthals.^38^ They also found the archaic haplotype at over 50% frequency in modern Papua New Guineans, which led them to suggest that this variant of the *STAT2* gene underwent positive selection in the ancestors of this population.^38^ Mendez et al. argued that *STAT2* represented the first confirmed case of *adaptive introgression*, where DNA sequences of archaic origin increase in frequency in a modern human population because they confer a selective advantage.^38^ However, it remains unclear what specific advantage the Neanderthal version of *STAT2* could have conferred on the ancestors of Papua New Guineans.

In recent years, this idea of modern humans acquiring beneficial genetic variants through introgression with archaic hominins has become a popular model for explaining how early human populations were able to rapidly adapt to the novel environments they encountered throughout the world.^44–50^ Huerta-Sanchez et al. analyzed the *EPAS1* haplotype in modern Tibetans, which is associated with adaptive physiological responses to the hypoxic (low oxygen) conditions typical of extremely high altitude environments.^51,52^ Using a panel of modern humans plus the Denisovan genome to perform a network analysis, which illustrates the relationships between haplotypes (see Box 5), Huerta-Sanchez et al. found that the Tibetan version of the *EPAS1* gene was most similar to the Denisovan.^51^ A subsequent network analysis conducted on a more comprehensive panel of modern humans showed that the Denisovan haplotype was also found in high altitude populations of the Himalayas, and clusters within a wide array of diverse African haplotypes that share many *EPAS1* alleles with the Denisovan.^53^ This broader context demonstrates that *EPAS1* haplotype variants were likely polymorphic in the ancestral human-Denisovan population and underwent incomplete lineage sorting (ILS) (see section on “Alternative explanations”) prior to introgression.^53^ Additionally, a follow-up study of modern Tibetan genomes found that their *EPAS1* haplotypes exhibit a combination of Denisovan and non-Denisovan variants.^54^ Based on these additional variants, the authors conclude that the population that contributed this haplotype to the ancestral Tibetan population had diverged from the reference Denisovan by between 238 and 952 ka.^54^ Further questions regarding the precise genetic basis of hypoxia adaptation and the timing of acquisition and selection on this archaic *EPAS1* haplotype in modern high-altitude populations continue to be investigated by both geneticists and archeologists.

An under-emphasized result of studies of the Neanderthal and Denisovan genomes is the lack of corroboration of genomic regions that had been previously hypothesized in earlier studies to be of archaic origin. For example, researchers suggested that the microcephalin (*MCPH1*) gene, which is involved in regulating brain volume, showed signatures of introgression.^43^ As in the previous examples, a long *MCPH1* haplotype bearing many derived substitutions was identified in 70% of individuals in a global panel but was at low frequency within Africans. However, no archaic specimen sequenced to date matches the candidate non-African version of *MCPH1*. This observation does not exclude the possibility that this haplotype has an archaic origin, as the genomes available for comparison represent only two of an unknown number of archaic species. They do, however, leave the status of *MCPH1* in question.

It is not clear how often these hypothesized archaic haplotypes are false positives, and to what extent they can be generated by other processes than introgression (see section on “Alternative explanations”). Furthermore, while network analyses can be very useful in visualizing the relationships between haplotypes, they can produce a biased picture when modern haplotype diversity is not adequately represented in the data set. Specifically, the lack of broad inclusion of samples from across the African continent often means that a broader context of AMH and archaic haplotype relationships is missed.

## 6 | HOW MUCH ARCHAIC INTROGRESSION OCCURRED?

**FIGURE 2:**
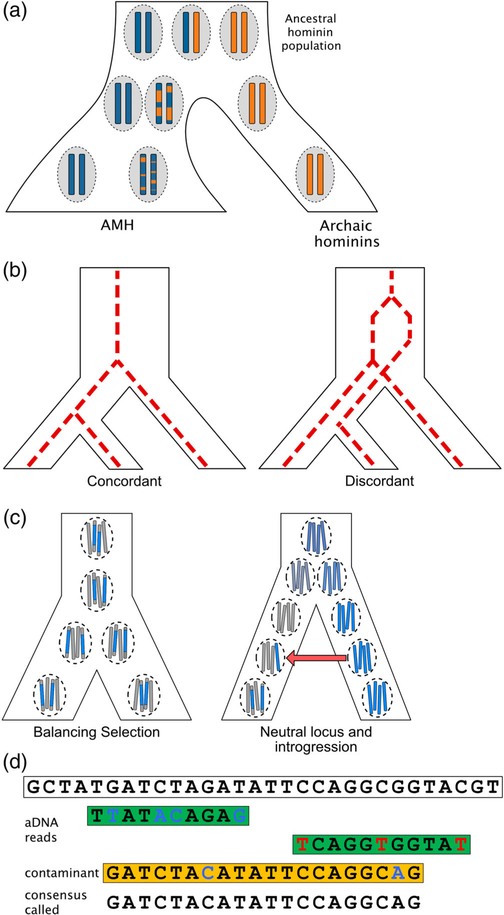
Illustrations of various alternative explanations that can often explain or bias genetic patterns that are interpreted as signatures of archaic introgression. Time progresses from top to bottom for all trees. (a) A structured ancestral population is comprised of two distinct ancestries (blue and orange) in distinct demes (dashed circles) that give rise to new demes over time. The two leftmost demes eventually give rise to AMHs, but one of them shares more ancestry with the deme that eventually gives rise to Neandertals and Denisovans. Due to recombination over generations, this ancestry is carried in the second AMH population in short tracts that are highly divergent in sequence from the blue ancestry carried by the first AMH population. This pattern occurs without needing to invoke post-population split introgression from the archaic hominin. (b) Different gene lineages within individuals and populations can have different evolutionary histories. A concordant gene lineage is one that conforms to the topology of the overall population tree. However, depending on the depth of divergence between the groups and the size of the ancestral population, some fraction of these lineages is expected to be affected by ILS. (c) Balancing selection can maintain highly diverged variants (blue and gray) of a specific genetic trait within a population (dashed circle) on long haplotypes over evolutionary time. Alternatively, if there is no selection acting to maintain variation at a locus, a long, highly diverged tract of ancestry could come from an archaic source. Recent introgression (red arrow) could bring this diverged ancestry into an AMH population, where it would lie on a long ancestral tract because relatively few generations of recombination have occurred. (d) A reference sequence (top) is used to align ancient archaic reads (green) from a sequencing experiment to recover the full sequence. Ancient DNA reads are typically short and contain a relatively high proportion of mismatches, either due to damage or diverged ancestry, compared with the reference. Observed C to T mutations (red) are due to a common form of DNA damage. Real mismatches (blue) can also occur because the archaic individual is usually substantially diverged from the reference, which is based on modern humans. Contaminant sequences from modern humans (orange), even if rare, can be favored by mapping algorithms because those fragments are longer and are more similar to the reference sequence. This leads to a reference-biased consensus sequence (bottom)

Studies have used various methods to estimate *f*, the average fraction of archaic ancestry that persists in modern individuals. In theory, it is a quantity that can simply be measured for any given genome. However, another important parameter for understanding human evolution is *m*, the migration rate. In population genetics, *m* is the proportion of migrant individuals contributing to a population in each generation. Archaic admixture is often modeled as a single pulse or a discrete event, making *m* the total proportion of an admixed human population that was comprised of archaic individuals. Under an extremely simplified demographic model that assumes no genetic drift, selection, or variation in hybrid and non-hybrid viability, the population’s average *f* after at least one generation would be equivalent to *m*. This is the model assumed by some influential articles (Table 2).^34,55^ However, the true relationship between *f* and *m* is certainly not this straightforward, and would instead depend on multiple factors such as the number and timing of introgression events, various demographic parameters, and the effects of selection, none of which are known with complete certainty (Figure 2). Therefore, the observed values of *f* in modern populations are consistent with a range of scenarios that involve relatively many to few individual hybridization events.

Neanderthal mtDNA provided the first archaic sequence data that could be used to address this question, and showed that that Neanderthal and modern human mtDNA gene pools were distinct and highly diverged. However, for reasons previously discussed, the absence of Neanderthal mitochondrial lineages in modern humans did not preclude the possibility of archaic introgression. Assuming that interbreeding did occur, Nordborg tested two admixture models to estimate the expected impact of Neanderthal mtDNA sequences on the extant human gene pool.^56^ Nordborg showed that, under the implausible scenario that AMH and Neanderthals comprised a single, randomly mating population, the observed mtDNA phylogeny would be highly unlikely.^56^ However, when considering a much more realistic model where some Neanderthal individuals were absorbed into a randomly mating modern human population, Nordborg showed that substantial levels of admixture could not be rejected (Box 2, Table 1).^56^ Specifically, if the hypothetical ancestral mtDNA pool was 25% Neanderthal, a much higher fraction than has been proposed in the literature, there was still a considerable chance (over 50%) that these archaic lineages would have gone extinct by the present (Table 1).^56^

This conclusion was subsequently challenged by Currat and Excoffier who argued that even this admixture model was overly simplistic.^24^ They used spatially explicit demographic models of the modern human range expansion into Europe, analogous to an advancing wave. The narrow, moving “wave front” represented the interaction zone where Neanderthals and modern humans could have potentially competed and/or interbred. Under this model, a Neanderthal mtDNA lineage has a higher chance of increasing in frequency when it enters an expanding population, such as at a wave front. These colonizers and their genes, including any acquired archaic component, would therefore enjoy a demographic advantage as their populations expanded to occupy new territory. Currat and Excoffier argued that this effect would result in even a small number of hybridization events having a disproportionate impact on the AMH gene pool.^24^ Therefore, they interpreted the absence of Neanderthal mtDNA lineages in extant humans as strong evidence that interbreeding between archaic and modern humans did not occur.^24^

Further studies explored additional demographic scenarios, each based on different models and assumptions, and inferring different values of *m* (Table 1). The availability of the nuclear genome provided new fodder to explore this topic. Initial analyses reporting an *f* of 1–4% seemed to demonstrate significant non-zero levels of migration from Neanderthals into AMH populations.^34^ In light of the Neanderthal nuclear data, Currat and Excoffier revisited their spatially explicit models, and found that a hybridization rate of less than 2% was compatible with the estimated levels of Neanderthal ancestry in modern humans, and concluded that the new observations were still compatible with strong reproductive isolation between Neanderthal and AMH populations and a complete lack of mtDNA sharing.^57^

In order to estimate *m* from whole genome data, Kuhlwilm et al. applied a Bayesian method to neutral stretches of sequence throughout the genome.^58^ By targeting regions that were less likely to be affected by natural selection, Kuhlwilm et al. estimated the initial migration fraction of Neanderthals into non-Africans to be 0.3–2.6% (Table 2).^58^ Harris and Nielsen, however, argued that neglecting to account for hybrid fitness could provide a skewed picture of patterns of Neanderthal ancestry in modern human genomes. Using simulations, they demonstrated that if Neanderthal-modern human hybrids exhibited higher fitness than modern humans, the average fraction of Neanderthal ancestry in modern humans could increase from an initial 1% to the currently observed approximately 3% within 500 generations (~15 ka) after introgression.^59^ However, if Neanderthal-human hybrids exhibited depressed fitness, an initial admixture fraction of 10% is compatible with current observations.^59^ Since the true fitness effects of Neanderthal variation on a modern human genetic background are not known, both scenarios and initial admixture fractions are plausible. Therefore, the persistence of a few fundamental uncertainties means that the initial level of gene flow between archaic and AM humans still cannot be known.

## 7 | WHERE AND WHEN DID ARCHAIC INTROGRESSION OCCUR?

Morphological arguments for admixture have long been made by paleoanthropologists, particularly those espousing regional continuity between AMH and preceding taxa. For example, Erik Trinkaus et al. have advocated for the hybrid status of several hominin fossils including those from Lagar Vehlo, Portugal^60^ and Peştera cu Oase, Romania.^61,62^ These proposed AMH-Neanderthal hybrids, which each represented a putative introgression event in a well-defined place and time, were not universally accepted. Many felt their apparent Neanderthal traits instead reflected shared ancestry or the wide range of intraspecific variation in *H. sapiens*.^63^

Green et al. were the first to use genetic evidence to note that Neanderthals were roughly equally related to Europeans as to East Asians and Papua New Guineans, suggesting that the main introgression event occurred between 50 and 80 ka when the ancestral Eurasian population resided in the Near East shortly after the Out of Africa migration.^34^ Subsequent studies have broadly agreed with that conclusion, while reporting increased precision on the date of gene flow. Sankararaman et al. estimated the date of gene flow between Neanderthals and modern humans to be 47–65 ka by analyzing patterns of *linkage disequilibrium* in extant genomes.^55^ Because *recombination* breaks apart pairs of alleles (and thus haplotypes), this process represents a type of molecular clock that tracks the number of generations that have passed since an introgression event; longer Neanderthal tracts in modern human genomes imply a more recent introgression event, and vice versa (Box 5).

Several directly dated fossil AMH which also carry Neanderthal ancestry suggest that gene flow must have occurred prior to 35 ka. Two AMH individuals, “Kostenki 14” dated to 36–38 ka in Russia and “Ust’ishim” 45 ka in western Siberia, carry Neanderthal haplotypes that are longer than the modern human average.^64,65^ These longer segments are expected when fewer generations (and thus, recombination events) have passed since the introgression event. Consistent with this timeline, the dates of Neanderthal-associated fossils and material culture indicate that they went extinct in Europe by approximately 40 ka, after overlapping with modern humans for several millennia.^66–68^ The aforementioned putative hybrid from Peştera cu Oase, Romania, dated to 37–42 ka, exhibits a Neanderthal haplotype pattern consistent with having a Neanderthal ancestor between 4 and 6 generations previously.^69^ However, this individual does not appear to have contributed significantly to the genetics of present-day humans, which is why this much later introgression time does not conflict with Sankararaman’s estimate.

Based on fossil evidence, the Neanderthal range lies completely outside of Africa, and many methods for inferring *f* rely on the assumption that sub-Saharan African populations carry no Neanderthal ancestry (see section on “Estimating the fraction of archaic ancestry in Modern Human Genomes,” Box 4). However, a recent article by Chen et al. found that in fact, the Neanderthal contribution to modern African genomes was not negligible. Using a new method for identifying Neanderthal sequence without relying on a “non-introgressed” reference population, the authors found that 0.3% of sub-Saharan African genomes was shared with Neanderthals.^70^ Importantly, they found that this was not due to primary admixture between the ancestors of modern sub-Saharan African populations and Neanderthals. Rather, this higher-than-expected level of Neanderthal sharing was driven by a combination of AMH migration back to Africa and by introgression of earlier AMH out-of-Africa migrants into Neanderthals prior to their extinction.^70^ In the latter scenario, the sequence sharing between Neanderthals and modern Africans can be explained by shared ancestral variation between the earlier out-of-Africa population, which admixed with Neanderthals, and the ancestral African population.^70^ It is unclear if and how much estimates of *f* will have to be revised in light of these findings (Table 2, Box 4), and if some methods are more robust than others.

The discovery of apparent Neanderthal DNA in sub-Saharan Africans also addresses one of the more puzzling findings from archaic aDNA: an apparent excess of Neanderthal-derived alleles in East Asian populations compared with Europeans, despite the absence of Neanderthal fossils in South or East Asia.^37,71,72^ Initial estimates suggested 40% more Neanderthal ancestry in the Han Chinese and Japanese, compared with Europeans.^75^ Neither the action of purifying selection nor changes in population size could be shown to explain this discrepancy, suggesting the possibility of additional Neanderthal introgression events into the ancestors of East Asians.^60,76^ However, in a comprehensive analysis of over 900 high coverage modern human genome sequences, Bergstrom et al. find evidence for only one major Neanderthal admixture event, noting minimal variation in Neanderthal haplotypes across all modern non-African populations.^37^ Chen et al. find that if the Neanderthal ancestry in modern Africans was introduced primarily by back-to-Africa migration from ancestral Europeans, their levels of Neanderthal ancestry would be systematically under-estimated relative to East Asians.^70^ Therefore, by accounting for the Neanderthal ancestry in modern Africans, Chen et al. find the discrepancy between estimates of *f* in Europeans and East Asians to be greatly reduced (Table 2).^70^

Inferring the timing and location of Denisovan introgression is an even more challenging problem given that there is little physical evidence of their presence, and genetic data from only a single cave in Siberia. Since the maximum levels of Denisovan ancestry have been identified in Melanesians, with significant Denisovan ancestry identified in Southeast Asian island and other Oceanian populations, it has been hypothesized that this archaic group ranged widely throughout Asia during the Late Pleistocene.^35,36^ This implies that the introgression event most likely occurred in the ancestors of Melanesians, presumably somewhere in continental Asia. Interestingly, there appears to be almost no Denisovan ancestry in modern or, based on one 40 ka old individual, ancient mainland Asian individuals, with the highest estimates being 0.1%.^36,73–75^ This could occur if there had been demographic turnover of the earlier mainland Asian populations, but not of Oceanians, that carried Denisovan ancestry.

Unlike for Neanderthals, the diversity of Denisovan haplotypes in humans suggests multiple introgression events from Denisovan-like hominins, and possibly even population structure between mainland Asian and insular haplotypes.^37,76,77^ Using the same linkage disequilibrium-based method as with Neanderthals, Sankararaman et al. estimated the date of Denisovan introgression to be 44–54 ka.^73^ Malaspinas et al. inferred a similar date of Denisovan introgression based on analysis of modern Aboriginal Australians and Papua New Guineans.^78^ Furthermore, by observing that the lengths of inferred Neanderthal haplotypes were significantly shorter than inferred Denisovan haplotypes in extant human genomes, both Malaspinas et al. and Sankararaman et al. concluded that Neanderthal introgression occurred prior to Denisovan introgression.^73,78^

## 8 | GENETIC INSIGHTS ON ARCHAIC FITNESS AND PHENOTYPE

While geneticists have characterized both genome-wide and fine-scale patterns of archaic admixture, there is a question of whether or not this admixture had any impact on fitness or phenotype. Phenotypic manifestations of admixture in skeletons are still poorly understood even for model organisms (but see the innovative comparative work of researchers such as Ackermann et al.^79–81^), which hampers conclusive morphological identification of hybrids in the fossil record. Therefore, while the true fitness of AMH–archaic hybrids is unknown, geneticists have attempted to estimate the strength of selection against archaic genetic contributions.

Taking a genome wide-perspective, Fu et al. analyzed aDNA from an AMH dating from 45,000–47,000 years ago and found that the proportion of Neanderthal DNA in AMH (*f*) declined from 3–6% to approximately 2%, implying strong selection against archaic genetic elements.^82^ However, a recent re-analysis of the data shows that the observed decline in *f* was an artifact of the statistic used in the original article, which failed to account for recent gene flow between modern human populations.^83^ Using an updated version of the statistic, they showed that the Neanderthal fraction in AMH has remained relatively steady at approximately 2.5% for over 40,000 years.^83^ However, they do find evidence for at least weak selection against Neanderthal genomic contributions to AMH genomes, in general agreement with previous studies.^83–85^ Theoretical work by Harris and Nielsen (discussed in section on “How much archaic introgression occurred?”) also supports the long term presence of archaic ancestry in AMH populations, even if there is initially strong selection against hybrids.^59^ They show that 10–20 generations of random mating within AMH would eventually drive variation in *f* across individuals to zero, diminishing the efficiency of selection against Neanderthal ancestry and leading to *f* becoming relatively stable over time.^59^

The question of how human modernity arose, and what genetic changes contributed to it, has been an active area of research for decades that is made even more complex by the possibility of archaic introgression. As discussed in the “Haplotype-Based Methods to Identify Genomic Regions of Introgression” section, locus-based putative cases of archaic adaptive introgression have been held up as evidence that “pre-adapted” elements of archaic ancestry facilitated modern human adaptation as they expanded into new habitats after leaving Africa.^38,44,47,86–88^ On the other hand, from the viewpoint of medical genetics and genome wide association studies, it has been argued that archaic introgression contributed variants that underlie several deleterious traits^89^ and has been directly selected against.^59,84^ While not mutually exclusive, these perspectives highlight another open question in the study of archaic introgression.^90^

In the early 2000s, the search for genetic signals of human behavioral modernity turned to the gene *FOXP2* and its role in complex language and cognition, a phenotype thought to differ between archaic and modern humans. When mutations in *FOXP2* were discovered in a family with high rates of severe speech and language impairment, it became the first gene candidate proposed to underlie human spoken language.^91^ In apparent support of its crucial importance to behavioral modernity, Enard et al. argued that *FOXP2* underwent a strong selective sweep recently in the modern human lineage that targeted two derived SNPs found in humans but not in chimpanzees.^92^ A selective sweep occurs when an advantageous allele arises in a population and then rapidly increases in frequency. Given their estimate of the timing of the putative sweep, Enard et al. hypothesized that FOXP2 had a key role in the evolution of human expression of complex symbolism and abstraction.^92^

With aDNA sequencing, however, these “human specific” variants were thrown into doubt when Neanderthals (and later, Denisovans) were shown to carry the same alleles as modern humans.^93^ Furthermore, several introgression studies found that this genomic region is notable for its lack of Neanderthal or Denisovan ancestry in modern humans.^70,73,87,94^ A re-analysis of *FOXP2* in a larger and more diverse genome-wide panel of modern individuals by Atkinson et al. was unable to replicate the previous finding of positive selection on *FOXP2* after explicitly accounting for human population demographic history.^95^ Instead, they showed that the pattern interpreted in Enard et al. to represent positive selection arose from a lack of global diversity in their dataset and confounding by population structure.^95^ These recent findings undercut the hypothesis that a recent selection sweep at *FOXP2* was critical for the evolution of advanced, *Homo sapiens*-specific, cognitive ability.^4^ Instead, in light of fine-scale genomic maps of archaic introgression, selection for the two derived SNPs had to have occurred in the common ancestral lineage of Neanderthals, Denisovans, and modern humans, and cannot account for the inferred behavioral differences between archaic and modern humans.

## 9 | ALTERNATIVE EXPLANATIONS

Claims of archaic ancestry in human genomes are generally made on the basis of characteristic patterns of variation observed in archaic and modern genomic data. However, as discussed throughout this review, other scenarios can also produce many of these signatures of introgression. As demonstrated by the early mtDNA studies, different demographic models can lead to different inferences of *m* from the same observed data (Table 1). Therefore, when reading the archaic genomics literature, it is important to pay careful attention to the assumptions made, assess whether these are reasonable, and to consider whether the reported observations could have arisen under a scenario lacking introgression.

Extreme values of a particular statistic are often reported as evidence for archaic introgression, especially in whole genome scans. What qualifies as “extreme” is generally based on a threshold set by the researcher. While some studies attempt to rigorously define extreme values by performing demographic simulations, choosing a realistic neutral model of human demography is not straightforward, and important factors, such as ancestral population structure, are often ignored for simplicity. Therefore, it is possible that an unusual parameter value under a simple (and unrealistic) neutral demographic model is not so extreme under a more realistic model. In this section, we outline other possible drivers of genomic signals resembling those that arise under archaic introgression (Figure 2).

### 9.1 | Ancestral population structure (non-random mating)

In studies of archaic admixture, ancestral human populations are often modeled as panmictic; that is, all members of the population choose a mate at random from among anyone else in the population. In reality, a multitude of factors (e.g., geography, language, and culture) structure populations such that certain pairings on individuals are much more likely than others. There is strong evidence from AMH morphology to suggest that the ancestral population was structured within Africa.^96–101^ A potential consequence of such structuring is that certain groups of modern humans might share more genetic variants with archaic hominins than others in the absence of recent introgression. Neanderthals, for example, could have split from the common ancestral population that later also gave rise to all non-African AMH populations (Figure 2a). Under this scenario, the observed excess of variants shared between Out of Africa individuals and Neanderthals would be due to ancient sharing of genetic lineages through persistent population structure over time.

The authors of an early Neanderthal genome study point out that they could not distinguish between ancestral population structure and archaic introgression.^34,102^ Indeed, Eriksson and Manica^103^ demonstrated that spatial structure in the ancestral hominin population could produce values of the D-statistic that were comparable with those obtained by Green et al., and were interpreted as evidence for archaic admixture.^34^ The degree to which ancestral population structure is responsible for the observed patterns of archaic ancestry is still debated.^104–107^ The presence of long tracts of archaic ancestry in extant non-African humans is the most convincing demonstration that their genetic similarity is driven by a recent introgression event and not ancestral population structure.^55^ However, the process of identifying regions that are shared between archaic and modern human genomes can be computationally challenging, with smaller (and older) tracts being more difficult to detect. It is, therefore, possible that ancestral population structure accounts for a significant proportion of the signal attributed to introgression.

### 9.2 | Incomplete lineage sorting

ILS refers to a discrepancy in the relationship between populations (or species) and genetic lineages. ILS and ancestral population structure are distinct concepts that can create similar patterns in genomic data. Over evolutionary time, both populations and genetic lineages generate trees through splitting and divergence (Figure 2b). In cases of relatively recently separated groups, such as Denisovans, Neanderthals, and AMH, a genetic lineage found in one individual can sometimes share its most recent common ancestor with an individual from the other group, even if each is panmictic.^107^ Therefore, some proportion of genetic lineages will be more recently shared between a particular human population and an archaic group by chance; this probability is proportional to the ancestral population size. As with ancestral population structure, the age of the shared variation is a distinguishing factor; if the archaic variant is on a long human haplotype, this is more indicative of recent admixture. Unlike ancestral population structure, however, ILS is not expected to generate more overall archaic sharing with one modern human group over another. Therefore, when looking across the entire genome, as in the D-test, the effect of ILS would theoretically be averaged out.

### 9.3 | Balancing selection

Balancing selection is a type of natural selection that maintains more than one haplotype in a population at intermediate frequencies over long time scales. This form of selection can occur if population-level variation improves fitness, as in the case of HLA genes related to immunity, if the environment is fluctuating, or if individual heterozygotes are more fit than either homozygote, as with sickle cell anemia in malarial environments.^108,109^ Unlike ancestral population structure and ILS, balancing selection can, under certain conditions, maintain ancient variation on long haplotypes, thus mimicking the signature of archaic admixture even more closely (Figure 2c). Specifically, longer than expected haplotypes can persist when there exist epistatic interactions between polymorphisms along its length, that is, the fitness of an allele depends on the presence of another allele some distance away.^110^ As a safeguard, regions that encode genes, and therefore might have been affected by balancing selection, are often excluded from analysis.^40^ However, this filtering greatly reduces power to identify biologically consequential cases of archaic introgression. Furthermore, it is difficult in practice to conclusively determine that a given region is not, or has never been, under balancing selection, even if it not near any genes. The possibility of balancing selection should therefore always be considered when studies purport to find evidence of adaptive introgression at a particular locus.

### 9.4 | Contamination

Contamination remains a problem in aDNA studies. Small amounts of modern contamination in archaic sequencing experiments can “modernize” ancient individuals, leading to incorrect inferences of population history and archaic admixture^28–30^ (Figure 2d). aDNA studies should always explicitly address the measures that were taken, both in handling and extracting the sample in the lab and in processing the sequence data, to measure and mitigate the effects of contamination.^111^

### 9.5 | Reference bias

When using a modern human reference to assemble genomes of highly diverged individuals, reference bias (or “mapping bias”) can occur. Reads in the sequencing library that are more similar to the reference are more likely to map, and thus be included in subsequent analyses (Figure 2d).^112^ Additionally, ancient fragments can be more difficult to map to a modern human reference because of sequence differences that are real (due to divergence) and/or artificial (due to DNA damage) (Figure 2d). This type of bias can also cause archaic genomes to look artificially similar to modern human genomes.^112^

### 9.6 | Ghost admixture

Recent evidence has highlighted the importance of ghost admixture, that is, introgression with populations for which there is neither descendant group nor even fossil evidence, in hominin evolutionary history. Certain features of the available genetic data of archaic and modern humans are best fit by population genetic models that include introgression events with as yet unidentified groups.^78,113^ Developing statistical methods to better detect the genetic signatures of introgression from ghost populations, for which there is by definition no reference genome, continues to be an active area of current research.^41,76,114,115^ Ghost admixture introduces complexities to population genetic models that are typically unaccounted for, especially in earlier studies of archaic introgression.

Rogers and Bohlender showed that estimators of *f* based on pairwise allele counts (such as the ratio of S-statistics) are prone to biases when introgression from ghost populations has occurred.^113^ The severity of this bias depends on how deeply diverged the populations in question are from each other.^113^ Rogers and Bohlender also found that different count-based estimators of the Denisovan contribution to Melanesians, based on a model of a single pulse of Denisovan introgression, are inconsistent with each other.^113^ They speculated that this may be due to a misspecification of the underlying demographic.^113^ Indeed, while early studies assumed a single introgression event in the ancestors of Melanesians, subsequent research has found evidence of multiple events from different Denisovan or Denisovan-like populations^37,76,77,116^ (see section on “Where and when did archaic introgression occur?”).

## 10 | CONCLUSIONS AND FUTURE DIRECTIONS

The field of aDNA and archaic introgression continues to rapidly expand as new specimens are sequenced, and novel laboratory and analytical techniques are developed. However, in the midst of these exciting advances, the statistical methods employed across studies often remain difficult to understand and to evaluate by non-specialists.^117^ In exploring the ever-burgeoning archaic admixture literature, it is prudent to pay careful attention to the details of these statistical tests, which are often relatively new and have been developed to accommodate the peculiarities and limitations of ancient data. Readers should always carefully note which assumptions are being made by the researchers, if these assumptions are reasonable, and consider the consequences of violating them for the overall conclusions. Alternative explanations for these patterns, some of which are outlined in this review, are often inadequately explored.

Given the sheer quantity of discoveries being made each year, it has not been possible cover all interesting facets of ancient introgression. Other recent reviews take complementary anthropological perspectives and dive deeper into many of the topics raised here.^46,118,119^ It will remain important that geneticists and paleoanthropologists continue to critically engage with, and evaluate, the findings of archaic introgression studies. In doing so, future multidisciplinary research will hopefully be able to address outstanding questions in the field, such as: What are the phenotypic effects of introgressed alleles in different human populations, and to what extent are surviving archaic alleles the result of adaptive introgression? How many introgression events occurred into AMH populations, what were the archaic sources, and what proportion of modern genomes is actually of archaic origin? This is still very much an open question given that estimates of *f*, the fraction of our genomes of archaic origin, have been constantly revised in light of new data (Table 2). Finally, we anticipate that ever larger and more diverse human genomic reference databases will enable the evaluation of more sophisticated hypotheses about how archaic admixture has impacted historically understudied populations in Africa, Asia, and the Americas.

## Figures and Tables

**TABLE 1:** Estimates of initial Neanderthal genomic contribution to AMH based only on mtDNA evidence

*m*	Model	Citation
<10%	Effective population size of AMH females is 16,000, and no archaic mtDNA is observed in a modern sample of 5,000 mtDNA sequences	^26^
Up to 25%	Single pulse, panmictic population	^56^
∼0%	No model, examined differences between mtDNA hypervariable regions of Neanderthals and AMHs (pairwise and in MDS space)	^22^
∼0%	Spatially explicit expansion of AMHs	^24^
Up to 24.3%	1 generation of AMH-Neanderthal coexistence	^14^
∼0%	400 generations of AMH-Neanderthal coexistence	
∼0%	Coalescent simulations of early, late, and no Neanderthal introgression	^25^

**TABLE 2:** Estimates of average Neanderthal admixture (*m* and/or *f*)

Proportion (*m* and/or *f*)	Method used	Citation
1.3–2.7% (non-Africans)	S-statistic	^34^
1–4% (non-Africans)	Parameterized population genetic model fit to D-statistics, introgression occurring 50–80 ka	
1.9–3.1% (non-Africans)	S-statistic	^35^
1–1.6% (Europeans) + 0.4–1.0% (“Eastern” non-Africans)	S-statistic	^36^
1.5–2.0% (Europeans)	S-statistic	^129^
1.6–2.1% (East Asians and Native South Americans)		
0.8% (Europeans and East Asians)	LD-based method^27^ and comparison to Neanderthal sequence	^49^
1.0–1.3% (Europeans)	Conditional random field-based model, combining allele matching, sequence divergence, and haplotype length information	^87^
1.3–1.5% (East Asians)		
1.0–1.3% (Native Americans)		
0.1–0.6% (Africans & African Americans)		
3.4–7.3% (non-Africans)	Likelihood maximization of parameterized demographic models	^105^
0.3–2.6% (non-Africans)	Bayesian approach using whole genome sequences (G-PhoCS)	^58^
0.9–1.2% (Western Eurasians)	Conditional random field-based model	^73^
1.3–1.5% (East and Central Asians)		
1.3–1.5% (Native Americans)		
1.1–1.3% (South Asians)		
1.4–1.7% Oceanians		
1.3–6.2% (non-Africans)	Site frequency spectrum analysis	^78^
0–1.3% (Oceanians)		
1.2% (Europeans)	Percentage of genome in putative archaic haplotypes	
1.4% (East Asians)		
1.2% (South Asians)		
1.4% (Native Americans)		
1.2–1.4% (Oceanians)		
1.8–2.4% (Western Eurasians)	S-statistic	^129^
2.3–2.6% (East Asians)		
2.1% (Western non-Africans)	F4 statistics	^37^
2.4% (Eastern non-Africans)		
0.8% (Europeans)	Percentage of genome in putative archaic haplotypes	^70^
0.9% (East Asians)		
0.9% (South Asians)		
0.3% (Africans)		

**TABLE 3:** Estimates of average Denisovan admixture (*m* or *f*)

Proportion (*m* and/or *f*)	Method used	Article
0% (Eurasians)	S-statistic	^35^
1.2–6.8% (Melanesians)		
3.8–5.8% (Melanesians)	Parameterized population genetic model fit to D-statistics	
0.8–6.2% (Oceanians)	S-statistic	^74^
0–2.4% (Southeast Asians)		
0% (South Asians)		
6% (Melanesians)	Inference of ancestral relationships using allele frequency data (TreeMix), without accounting for Neanderthal admixture	^36^
2.2–3.8% (Melanesians)	S-statistic	
<0.1% (East Asians)		
2.3–3.7% (Melanesians)	Bayesian approach using whole genome sequences (G-PhoCS)	^58^
0.1–1.6% (East Asians)		
0.8% (Oceanians)	Conditional random field-based model	^73^
1.9–3.4% (Melanesians)	F4 statistics	^94^
3.3–5.0% (Oceanians)	Site frequency spectrum analysis	^78^
0%(Europeans)	Percentage of genome in putative archaic haplotypes	
0.1% (East Asians)		
0.1% (South Asians)		
0.1% (Native Americans)		
0.2–1.2% (Oceanians)		
2.8% (Oceanians)	F4 statistics	^37^

## Data Availability

Data sharing is not applicable to this article as no new data were created or analyzed in this study.

## BOX 1 Glossary

Adaptive introgression: The movement of genetic variants from one population or species into another that provide an evolutionary advantage to the population or species that acquires them.

Allele: One version of a genetic polymorphism. For example, consider a single position in the genome that is variable in a sample between adenine (A) and thymine (T) nucleotides. A and T are referred to as the variant’s two alleles.

Coverage: The number of times a specific genomic position has been independently sequenced, or a genome-wide average of this value across all positions for a given sequencing experiment.

Effective population size: The hypothetical number of individuals that a population would need to contain in order to exhibit certain characteristics under an idealized model. This can be estimated from the amount of genetic diversity in a sample, among other features. For example, a population can have a census size of 9,000 but exhibit a low level of genetic diversity expected of an idealized population of only 3,000 individuals. Thus, this hypothetical population’s census population size is three times that of its effective size.

Genetic drift: The random change in allele frequency over time in a population due to stochastic variation from one generation to the next. The power of genetic drift on a population’s evolutionary trajectory is inversely correlated with its effective population size.

Haplotype: A group of alleles that tend to be inherited together from one generation to the next due to their physical linkage on a chromosome.

Linkage disequilibrium: The statistical association between alleles at different positions in the genome, generally due to physical proximity and genetic linkage between loci.

Locus (plural loci): A specific region of the genome.

Nuclear genome: The genetic information contained within the nucleus of the cell. In humans, it is comprised of 22 autosomal chromosomes and two sex chromosomes (X and Y). Because of recombination, which occurs on all chromosomes except the Y, an individual’s nuclear genome represents thousands of independent loci that are informative about historical demographic processes.

Mapping: The process of computationally aligning sequencing reads to a reference genome.

Mitochondrial genome: A small circular piece of non-recombining DNA that is contained within the mitochondria of the cell. It is transmitted from a mother to all her offspring (see Box 2).

Population divergence: The process by which distinct populations become more genetically distinct from each other over time. This can occur entirely due to neutral processes (genetic drift and mutation) acting over generations.

Population split: The point in time when two populations, derived from one larger ancestral population, cease to be governed by the same evolutionary processes. Once populations split, they continue to diverge over time.

Recombination: The process by which chromosome pairs exchange segments during the formation of gametes. When it occurs normally, no information is gained or lost from the full complement of chromosomes, but new configurations of genetic variants can arise. In the parent, the grandparental chromosome pairs can recombine with each other, resulting in a recombinant chromosome that is passed down to the parent’s offspring.

Sequence divergence: The process by which genetic lineages accumulate mutational differences from each other over time. Sequences can diverge independent of population divergence or splitting.

Single nucleotide polymorphisms (SNPs): Genetic variants in the identity of a single base in the genome.

## BOX 2 Mitochondrial DNA

The human *mitochondrial genome* is a small (16,569 bp) stretch of non-recombining DNA that is passed from mother to child through the mother’s egg cell. Over evolutionary time, mutations accumulate in different mitochondrial lineages, which makes it possible to reconstruct past relationships between different groups and trace the genetic history of females in the population. The pedigree figure above left, which depicts females as circles and males as squares, shows the transmission of mitochondrial genomes (colored ovals) through the generations. Without *recombination*, offspring carry the same sequence as their mother, except when novel mutations occur.

Early aDNA studies found that, in old, degraded specimens, mitochondrial sequences were the most readily recoverable DNA/genetic material. This is primarily due to their abundance; each cell in the body carries only two copies of the *nuclear genome*, but up to thousands of mitochondria that each contain several copies of their genome. Furthermore, because the modern human mitochondrial sequence was well known, it was feasible to target a phylogenetically informative region in an ancient specimen for sequencing using the older Sanger sequencing technology. For these reasons, mitochondrial aDNA quickly became an important source of information for studies of archaic admixture.

However, mtDNA has limited power to conclusively answer whether or not archaic and AMH interbred. One reason is that, because it is only transmitted through the generations by females, the mitochondrial genome always has a smaller *effective population size* than the autosomal nuclear genome and is subject to a proportionately stronger degree of *genetic drift*. Therefore, while the absence of archaic mtDNA lineages in modern humans was interpreted by some to indicate no introgression, this observation is in fact compatible with a substantial level introgression. This situation is illustrated in the figure above right, where introgression occurs at generation *t*_1_ with a small number of yellow Neanderthal mitochondrial sequences migrating into the modern human gene pool (red arrow). Over the next few generations, the frequency of the yellow mitochondrial eventually drifts to zero even in the absence of negative selection.

It is important to note that this illustration depicts only one possible iteration of the highly stochastic process that leads to new generations. Under this model, it is also possible that the yellow type persists in the human gene pool until the present day. The likelihood of this scenario increases with higher levels of initial migration (*m*), and decreases with the age of the gene flow event. In order to determine how many independent *loci* (i.e., different iterations of the evolutionary process) would be needed to make a determination on the occurrence of admixture between modern humans and Neanderthals, Wall conducted a power analysis assuming a specific demographic model, and estimated that information from 50 to 100 independent loci would be needed.^137^ Therefore, while mitochondrial sequence information can paint a general picture of the evolutionary relationship between populations, it offers inadequate resolution to rule out low levels of archaic introgression.

## BOX 3 Early Neanderthal genome studies

In 2006, the first two studies of a nuclear Neanderthal genome published significant quantities of ancient sequence and also inferred population genetic parameters such as Neanderthal-AMH divergence time and relatedness.^27,28^ It was immediately clear that these two studies had inconsistent estimates of these fundamental parameters, motivating further analyses to understand the drivers of these discrepancies.^29,30^

Noonan et al. estimated a human-Neanderthal DNA sequence divergence date of 706 ka, while Green et al. estimated a date of 516 ka, with very little overlap in their 95% confidence intervals. Additionally, Green et al. estimated a divergence time between modern human sequences that overlapped considerably with their estimate of the human–Neanderthal divergence time. This result in particular should have prompted skepticism from the research team, considering the hominin fossil record, as well as previous mtDNA studies, which estimated the human-Neanderthal divergence time to be 3–4 times older than the divergence among modern human sequences^15,16^ (Figure 1).

By surveying a set of *single nucleotide polymorphisms (SNPs)*, Green et al. also claimed evidence for substantial levels of Neanderthal introgression.^28^ Specifically, the researchers focused on genomic positions that were variable in a modern human panel. They inferred a polymorphism’s ancestral *allele* by looking at the homologous site in the chimpanzee genome. Green et al. found that Neanderthals shared the derived allele with some modern humans at far more loci than they would have expected under a simple demographic model.^28^ They concluded that this excess of derived SNP sharing was due to the occurrence of archaic introgression into some ancestral human populations.^28^ By contrast, the study by Noonan et al. found no evidence of introgression; they surveyed their data for derived alleles that were at low frequency in Europeans and were also shared with the archaic individual, and found none.^27^

In a reanalysis of both datasets and using a uniform set of methods, Wall and Kim confirmed large inconsistencies between both studies.^29^ From the Green et al. dataset, Wall and Kim estimated the modern European–Neanderthal *population split* time to have been extremely recent (35 ka), while from the Noonan et al. dataset they inferred a much earlier date (325 ka). Furthermore, they found that the degree of sequence similarity between the Green et al. Neanderthal and modern Europeans suggested an extremely high Neanderthal admixture proportion of 94%.

Strikingly, Wall and Kim also found that their estimate of the human-Neanderthal divergence time from the Green et al. dataset changed depending on the length of the sequenced DNA fragment considered; using only short sequencing reads, their estimate was 696 ka, but the medium and large read classes yielded significantly younger divergence times.^29^ Since aDNA is highly fragmented, authentic archaic sequences would most likely fall in the shortest read class, while any contaminating modern sequences would tend to fall in the longer read classes. As a result, Wall and Kim ultimately concluded that the Green et al. dataset had been significantly contaminated by a modern human.

## BOX 4

$$D=\frac{\sum_{i=1}^{n}\left[C_{ABBA}(i)-C_{BABA}(i)\right]}{\sum_{i=1}^{n}\left[C_{ABBA}(i)+C_{BABA}(i)\right]}$$

The D statistic was first used by Green et al. (2010) to demonstrate that Neanderthals appeared to be more similar to non-African modern humans than Africans.^34^ The appeal of this statistic, and its subsequent widespread use, can be attributed to its simplicity and the fact that it can be calculated even when there is only a single haplotype representing the archaic population. As illustrated above, the D statistic compares the number of derived *alleles* shared between the archaic specimen (N) and one of the modern human populations but not the other (H1/H2) at biallelic sites that exhibit either an “ABBA” or “BABA” pattern. These are determined through comparison to an outgroup, in this case the chimpanzee (Pan). The chimpanzee state is assumed to be ancestral, and is denoted as “A,” while the derived allele is denoted as “B.” While this assumption may not always hold, such as in the case of recurrent mutations on the chimpanzee lineage, the effects of this type of misspecification are not expected to systematically bias this statistic, as long as mutation rates across human groups are constant.^102^ Multiple *loci* are tested for “ABBA” and “BABA” patterns, which do not follow the population tree and are thus expected to be a result of either introgression, *ILS*, or recurrent mutation. As the latter two processes are expected to affect all human populations equally, they should generate as many ABBA *single nucleotide polymorphisms (SNPs)* as BABA SNPs. In the equation on the left, c is either 1 or 0 based on whether the pattern is seen or not. To calculate D, the number of sites that conform to the ABBA pattern is subtracted from the number that conform to the BABA pattern and divided by the total number of sites considered. Thus, values of D that significantly deviate from 0 (ABBA–BABA equality) can support the presence of introgressed archaic ancestry in one of the modern populations.

$$f\approx \frac{S\left(H_{Afr},H_{nAfr},N,Pan\right)}{S\left(H_{Afr},N_{A},N,Pan\right)}$$

Importantly, the D-statistic does not directly yield an estimate of the archaic ancestry proportion (*f*), but is simply an observation that parameterized demographic models can be compared with. Another method of obtaining a point estimate of *f* uses the S-statistic, which is simply the numerator of the D-statistic. The equation and diagram below and right show how, in theory, the ratio between S-statistics can be used to estimate *f* directly, where H_nAfr_ is a modern non-African human population whose ancestors experienced introgression, H_Afr_ is the African population that is assumed to have not experienced introgression, and N_A_ is the ancestral Neanderthal population that contributed genetic material to the ancestors of H_nAfr_. In practice, N_A_ cannot be known for certain, so a second Neanderthal individual is used as a proxy. The numerator measures how much more similar the first Neanderthal is to the modern non-African than to the modern African. The denominator yields an estimate of the maximum value of S when comparing two Neanderthals. By normalizing the observed level of sharing between non-Africans and Neanderthals by this theoretical maximum, this ratio infers the proportion of the observed similarity that is due to introgression.

However, recent work by Chen et al. invalidates the assumption that Africans carry negligible Neanderthal ancestry, which is often made in estimating *f* in non-Africans using S-statistic ratios of the above form.^70^ The presence of excess derived allele sharing between Africans and Neanderthals due to introgression may bias estimates of *f* in non-Africans by reducing the numerator S-statistic. The magnitude of this effect would depend on what proportion of the African-Neanderthal sharing is also shared by the non-African population; this would decrease the number of sites available to calculate the S-statistic, but should not downwardly bias *f*. Interestingly, 94% of the Neanderthal ancestry in Africans is also shared with a non-African group.^70^ African-Neanderthal sharing could also inflate estimates of *f* in non-Africans by shrinking the denominator of the above equation. This would happen if there is Neanderthal population structure such that the African population analyzed shared more derived alleles with N than N_A_. Therefore, in light of the recent findings by Chen et al., further simulation-based analyses will be necessary to understand how prior S-statistic-based estimates of *f* should be interpreted and amended. This is underscored by previous work by Rogers and Bohlender that shows that S-statistics are sensitive to violations of the underlying population genetic model, such as when unaccounted for ghost admixture has occurred (see section on “Alternative explanations”), leading to large biases in *f*.^113^

While model misspecification continues to be problematic, D and S-statistics can be useful in cases where there is limited genomic data from the putative introgression source, and have been widely used in other contexts since they were introduced. In using a set of unascertained SNPs, they can produce a broad picture of the degree of genetic similarity between individuals. They are meant to be used only as genome-wide measures; for any particular locus, it is possible that other factors unrelated to introgression, such as recurrent mutation and ILS, as well as low effective population size and low locus-specific genomic diversity, could result in extreme D or S values.^34,102,138^

## BOX 5

A *haplotype* is a specific combination of *alleles* at *loci* that lie close together along a chromosome. Because of this physical proximity and linkage, the individual variants composing a haplotype tend to be inherited together. Three distinct haplotypes comprised of six alleles each are depicted above, with the dark bar representing the intervening sequence that is shared between all of them. At each variable position, a haplotype can carry one of two alleles. Along with the variants themselves, the associations between them provide information about demographic history and evolutionary processes. Haplotypes are passed down from parent to offspring with *recombination* between the parent’s chromosomes. Both mutation and recombination affect haplotype patterns in a generation time-dependent manner, making them useful for inferring parameters related to archaic introgression, including the extent and timing of gene flow between groups.

Whenever recombination occurs, it disrupts the continuity of the haplotype. Because recombination occurs at a particular rate per generation, distinct haplotypes are expected to break down steadily over time. Therefore, haplotype length can be used to approximately date introgression events.^139^ As shown in the figure (left), in the first generation after gene flow, the hybrid offspring would have a full complement of AMH (green) and archaic (purple) chromosomes. With each subsequent generation, pieces of the introgressed chromosome are shuffled by recombination (red arrows) into an AMH genetic background and eroded by successive recombination. This would eventually lead to individuals in the population carrying their archaic ancestry in small tracts. With archaic genomes, it is possible to identify autosomal haplotypes in modern humans that approximately match either Neanderthals or Denisovans. It is assumed that these extended matching haplotypes entered the human gene pool via archaic introgression; the shorter the shared haplotype, the more recombination has occurred and the older the introgression event.

Haplotype divergence is another feature that can be used to estimate the relative age of a genomic segment. Because mutations also occur at a particular rate per generation, the number of nucleotide differences between two haplotypes reflects their evolutionary distance. In the figure above, the colored blocks in the sequence which are not yellow represent only the variable positions of the haplotype. Some of these haplotypes are passed down to the next generation with the occasional mutation. With each generation, the diversity of the set increases as the haplotypes become more different from each other. Haplotypes within the two populations are more similar that the ones between populations. AMH carry some haplotypes that are unusually diverged from the rest, given our relatively recent origin. Archaic introgression is often invoked to explain this pattern, since gene flow will carry haplotypes from one population into another. Additionally, the worldwide pattern of haplotype variation can support an introgression hypothesis for a particular locus. For example, given the geographic range of Neanderthals, it is unlikely that the ancestors of sub-Saharan Africans would have interbred with them. Therefore, a highly diverged haplotype that is common in Europeans and is highly uncommon in sub-Saharan Africans is consistent with being of Neanderthal origin. In cases where genomic data from the hypothesized archaic source exists, it is also possible to compare their sequences and determine if the haplotypes are closely related. However, the extreme lack of representation of modern Africans in genetic databases may be biasing this view—basal haplotypes that are assumed to be absent in Africa may simply be unsampled there as of yet.

A common way that haplotypes are represented is through haplotype networks, illustrated below. The nodes represent groups of haplotypes that are identical, and their sizes are proportional to the number of haplotypes they contain. The edge lengths represent the number of genetic differences from that node to the next most closely related one. The nodes are usually colored by the population that the haplotype was sampled from. For example, the leftmost network has a large node that contains multiple colors, representing a single haplotype that is shared across populations 1–4. The network in the middle shows a locus where haplotypes are highly population specific. All the haplotypes have a common origin, which is carried mostly by individuals belonging population 1, and to a much lesser extent, populations 2 and 4. The rightmost network shows a haplotype that has a high degree of differentiation among samples, with many unique haplotypes that are only slightly different from the haplotype of origin.
